# Effects of carbonated drinks, alcoholic drinks and mouthwashes with varying pH on mechanical properties and colour stability of various thermoplastic retainers, aligners and occlusal splints: an in-vitro study

**DOI:** 10.1590/2177-6709.31.1.e2625178.oar

**Published:** 2026-04-17

**Authors:** Mahulkar NIHARIKA, Vizia MUDDADA, Navya PUVVULA, Payal Jyoti DASH, Limasunep JAMIR, Udayadityeshwari GALI, Bandela Anand BABU, Pentapalli PRASANTHI

**Affiliations:** 1 Sree Sai Dental College and Research Institute, Department of Orthodontics (Chapuram, Srikakulam, India).; 2 Deemed-to-be University, Kalinga Institute of Industrial Technology, Kalinga Institute of Dental Sciences (Bhubaneswar, Odisha, India).

**Keywords:** Orthodontics, clear aligners, Thermoplastic materials, Physical properties, Colour stability, Ortodontia, alinhadores transparentes, Materiais termoplásticos, Propriedades físicas, Estabilidade da cor

## Abstract

**Introduction:**

Thermoplastic materials used for retainers, aligners, and occlusal splints may degrade when exposed to common beverages and mouthwashes, affecting mechanical properties and aesthetics.

**Objective:**

To evaluate the effects of carbonated drinks, alcoholic beverages (brandy), and mouthwashes on the mechanical properties and colour stability of various orthodontic thermoplastics.

**Material and Methods:**

A total of 144 samples from six thermoplastic materials (Erkodur, Duran, Erkodur-Al, CA Pro+, Erkoflex, Bioplast) were immersed daily for 10 minutes over 14 days in artificial saliva, brandy, carbonated drink, or chlorhexidine mouthwash. Mechanical properties (yield strength, stiffness, elastic modulus) were measured via universal testing machine; colour changes (ΔE) were assessed with a colorimeter. One-way ANOVA was used for statistical analysis (p < 0.05).

**Results:**

Retainers immersed in saliva exhibited highest yield strength (Erkodur: 61.95 ± 4.21; Duran: 59.25 ± 3.12) and stiffness (Erkodur: 2.12 ± 0.19; Duran: 2.12 ± 0.23), with lowest values in carbonated drinks (Erkodur: 48.88 ± 3.32 ; Duran: 48.45 ± 5.77). Aligners and occlusal splints followed similar trends; Bioplast splints performed best mechanically (saliva: 63.05 ± 4.36 ; stiffness: 1.98 ± 0.13). Colour changes were greatest in alcoholic beverages, notably Duran (ΔE: 0.978 ± 0.21, p = 0.010) and Erkoflex (ΔE: 1.21 ± 0.06, p = 0.003); aligners and Bioplast showed minimal ΔE differences.

**Conclusion:**

Acidic and alcoholic beverages significantly reduce mechanical strength and compromise aesthetics of thermoplastic orthodontic appliances. Bioplast exhibited superior mechanical performance among splints, while aligners demonstrated minimal discoloration. Patient education on limiting exposure to such beverages is crucial to maintain appliance durability and optimal treatment outcomes.

## RESUMO

**Introdução:**

Os materiais termoplásticos usados em aparelhos de contenção, alinhadores e placas oclusais podem se degradar quando expostos a bebidas comuns e enxaguantes bucais, afetando suas propriedades mecânicas e estéticas.

**Objetivo:**

Avaliar os efeitos de refrigerantes, bebidas alcoólicas (conhaque) e enxaguantes bucais nas propriedades mecânicas e na estabilidade da cor de diversos materiais termoplásticos de uso ortodôntico.

**Material e Métodos:**

144 amostras de seis materiais termoplásticos (Erkodur, Duran, Erkodur-Al, CA Pro+, Erkoflex, Bioplast) foram imersas diariamente por 10 minutos, durante 14 dias, em saliva artificial, conhaque, refrigerante ou enxaguante bucal com clorexidina. As propriedades mecânicas (limite de escoamento, rigidez, módulo de elasticidade) foram medidas por meio de uma máquina de ensaio universal; as alterações de cor (ΔE) foram avaliadas com um colorímetro. A análise estatística foi realizada com o teste ANOVA de uma via (p < 0,05).

**Resultados:**

Os aparelhos de contenção imersos em saliva apresentaram o maior limite de escoamento (Erkodur: 61,95 ± 4,21; Duran: 59,25 ± 3,12) e rigidez (Erkodur: 2,12 ± 0,19; Duran: 2,12 ± 0,23), com os menores valores observados em refrigerantes (Erkodur: 48,88 ± 3,32; Duran: 48,45 ± 5,77). Os alinhadores e as placas oclusais seguiram tendências semelhantes; as placas de Bioplast apresentaram o melhor desempenho mecânico (saliva: 63,05 ± 4,36; rigidez: 1,98 ± 0,13). As alterações de cor foram maiores nas bebidas alcoólicas, principalmente nos grupos Duran (ΔE: 0,978 ± 0,21, p = 0,010) e Erkoflex (ΔE: 1,21 ± 0,06, p = 0,003); os alinhadores e o Bioplast apresentaram diferenças mínimas de ΔE.

**Conclusão:**

Bebidas ácidas e alcoólicas reduzem significativamente a resistência mecânica e comprometem a estética dos aparelhos ortodônticos termoplásticos. O Bioplast apresentou desempenho mecânico superior entre as placas de contenção, enquanto os alinhadores demonstraram mínima descoloração. A educação do paciente sobre a importância de evitar a exposição a essas bebidas é crucial para manter a durabilidade do aparelho e obter resultados ideais no tratamento.

## INTRODUCTION

Since the era of Edward Hartley Angle, the father of modern orthodontics, there has been a transformative evolution in treatment modalities. The growing demand for discreet and comfortable orthodontic care, especially among adults, has led to the rapid popularity of clear aligners.^1^ ^-^ ^5^

Clear aligners, though widely recognized today through commercial systems like Invisalign^®^, have their roots in earlier concepts such as Kesling’s tooth positioner and Nahoum’s vacuum-formed appliances. Modern aligners utilize CAD/CAM technology and 3D printing, offering better precision, customization, and biomechanical control. Their aesthetic appeal, ease of use, and removability make them a preferred choice for both clinicians and patients. Alongside aligners, thermoplastic retainers and occlusal splints have also gained widespread use for their transparency, durability, and patient compliance.^6^ ^-^ ^8^

Despite these advantages, the long-term performance of thermoplastic appliances can be compromised by the oral environment. These materials are continuously exposed to fluctuating temperature, salivary enzymes, and a variety of dietary substances. Beverages such as carbonated drinks, alcoholic beverages, and oral hygiene products like mouthwashes often possess varying pH levels and chemical compositions that may alter the structural integrity and color stability of orthodontic materials. Prolonged exposure to these substances can lead to reduced mechanical properties such as yield strength, stiffness, and elastic modulus, as well as aesthetic deterioration through discoloration or opacity.^9^ ^-^ ^11^

Given the increasing use of these appliances and their daily exposure to potentially degrading agents, it is important to investigate how such factors affect their performance. A thorough understanding of these interactions will aid in material selection, patient education, and improved treatment outcomes. Therefore, this study was designed to evaluate the effects of commonly consumed beverages and commercially available mouthwashes - with differing pH levels - on the mechanical strength and aesthetic properties of thermoplastic materials used in clear aligners, retainers, and occlusal splints. 

## MATERIAL AND METHODS

### SAMPLE SIZE CALCULATION

An a priori sample size calculation was performed using G*Power for a one-way ANOVA (fixed effects, omnibus, one-way). The calculation was based on an effect size of *f* = 0.264, α = 0.05, and a desired power (1−β) = 0.80. The analysis indicated that a total of 144 samples would be required to achieve adequate statistical power (Actual power = 0.808), distributed as 48 samples per main group, 24 samples per subgroup, and 6 samples per sub-subgroup.

A total of 144 thermoplastic samples were prepared and categorized into three main groups: retainers, aligners, and occlusal splints. Each group comprised 48 samples, further subdivided into two commercial brands (n = 24 each), and subsequently into four immersion subgroups based on the solution used: artificial saliva (pH 6.8), brandy (pH 3.5), carbonated beverage (Thums Up, pH 2.68), and chlorhexidine mouthwash (pH 5.5-7).

### ARTIFICIAL SALIVA PREPARATION

Fusayama-Meyer’s artificial saliva was prepared according to the formulation described by Fusayama et al.^12^ The composition was as follows: NaCl (0.400 g/L), KCl (0.400 g/L), CaCl_2_·2H_2_O (0.906 g/L), NaH_2_PO_4_·2H_2_O (0.690 g/L), Na_2_S·9H_2_O (0.005 g/L), and urea (1.0 g/L). All reagents were of analytical grade and dissolved in distilled water under constant magnetic stirring until complete dissolution. The solution was freshly prepared before use and had a pH of 6.8 ± 0.1, simulating near-physiological oral conditions. 

### MATERIALS USED

1. Retainers:
  » Erkodur^®^ (Erkodent Erich Kopp GmbH, Germany; 0.8 mm; PET-G).
  » Duran^®^ (Scheu-Dental GmbH, Germany; 0.75 mm; PET-G).
2. Aligners:
  » Erkodur-Al^®^ (Erkodent Erich Kopp GmbH, Germany; 0.8 mm; Copolyester).
  » CA^®^ Pro+ (Scheu-Dental GmbH, Germany; 0.75 mm; multilayer with outer copolyester and inner thermoplastic elastomer).
3. Occlusal splints:
  » Erkoflex^®^ (Erkodent Erich Kopp GmbH, Germany; 1.5 mm; EVA).
  » Bioplast^®^ (Scheu-Dental GmbH, Germany; 1.5 mm; EVA).

### THERMOFORMING AND SAMPLE PREPARATION

**Figure 1:**
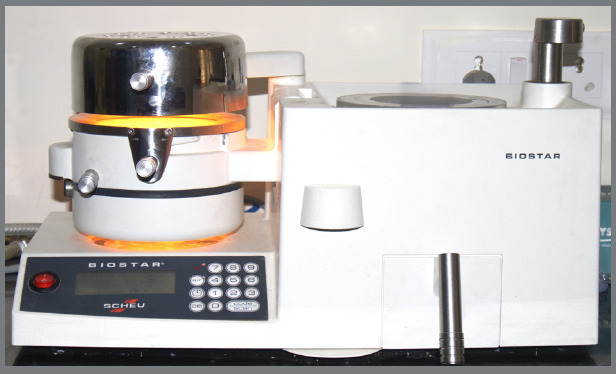
Biostar thermoforming machine used for fabrication of thermoplastic specimens.

**Figure 2:**
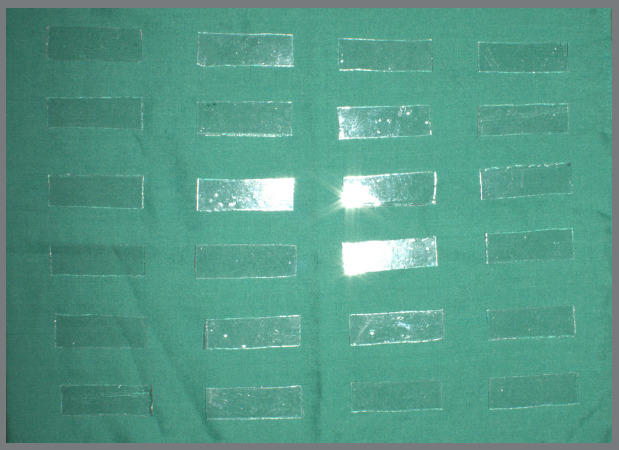
Rectangular thermoplastic specimens (20 × 60 mm) obtained after thermoforming.

Thermoplastic sheets were thermoformed using a Biostar machine (Fig. 1) over a 10-mm flat dental stone block. Rectangular specimens (20 mm x 60 mm) were cut from the thermoformed sheets (Fig. 2).

### AGING PROTOCOL

Samples were immersed daily in the four solutions for 10 minutes, over 14 consecutive days. After exposure, they were rinsed in distilled water and dried at room temperature.

The samples were immersed in the test solutions for 10 minutes per day, to simulate occasional exposure during social events when removal is not possible. Between exposures, samples were stored in artificial saliva at room temperature. While this does not reflect continuous wear, it provides a controlled, repeatable method to assess material changes in a clinically relevant scenario.

## SAMPLE ALLOCATION AND TESTING PROCEDURE

The allocation of samples to each immersion subgroup was performed systematically, to ensure equal distribution (n = 6 per solution). After the exposure period, all specimens were coded and sent to an independent laboratory for testing. The laboratory personnel performing the mechanical (UTM) and color (FRU Colorimeter) analyses were blinded to the sample grouping, to prevent observer bias. This ensured objective and consistent measurement across all samples.

### MECHANICAL TESTING

**Figure 3:**
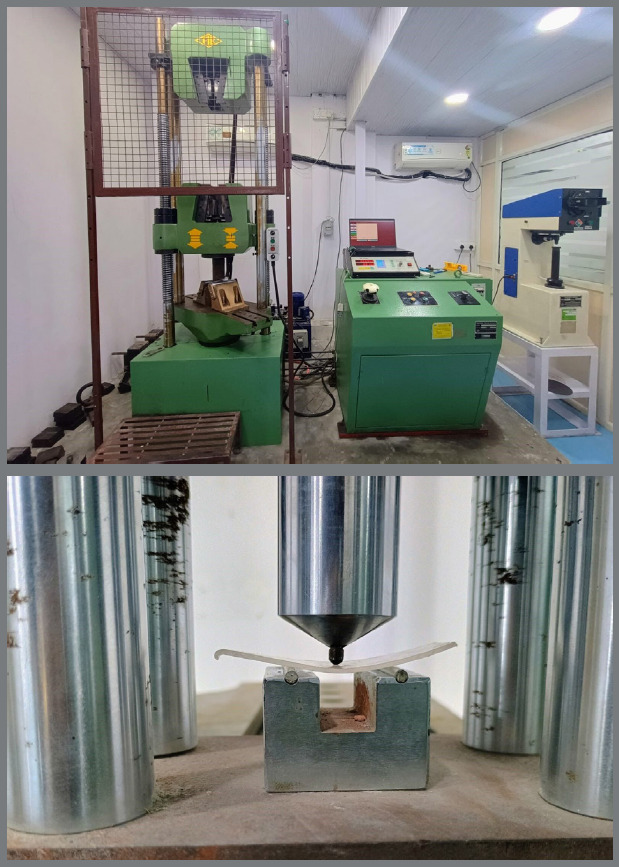
Universal testing machine setup for three-point bending test.

Yield Strength, Stiffness, Elastic Modulus: Evaluated using a Universal Testing Machine with a 3-point bending test setup (Fig. 3).

### COLOUR STABILITY ASSESSMENT

**Figure 4:**
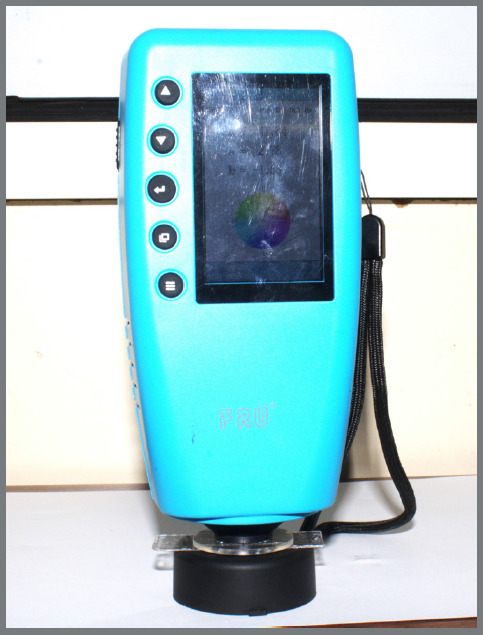
FRU WR Series Colorimeter used to measure colour change (ΔE) values using the CIELAB system.

Colour stability was measured using FRU WR Series Colorimeter using the CIELAB (Lab*) system (Fig. 4) Colour change (ΔE) was calculated after exposure. In this study, the term *colour stability* is used in the title and discussion; however, since ΔE directly measures colour change, it is important to note that ΔE values are inversely proportional to colour stability (i.e., higher ΔE indicates lower stability).

Statistical analysis was performed using one-way ANOVA with significance set at p < 0.05.

## RESULTS

Intragroup comparisons using one-way ANOVA revealed that for all retainer (Erkodur, Duran), aligner (Erkodur-Al, CA Pro+), and occlusal splint (Erkoflex, Bioplast) materials, immersion in saliva consistently resulted in the highest mean values for yield strength, stiffness, and elastic modulus, while immersion in carbonated drinks showed the lowest values across all parameters, with statistically significant differences (p < 0.05) in each group. Among retainers, Duran showed slightly higher mechanical properties than Erkodur; among aligners, CA Pro+ performed better than Erkodur-Al; and among splints, Bioplast showed superior values compared to Erkoflex. Regarding color stability, the greatest color changes were observed in samples immersed in alcohol or carbonated beverages, though statistical significance was observed only in selected groups - specifically Duran (p = 0.010) and Erkoflex (p = 0.003). Aligners and Bioplast showed no statistically significant differences in color change among subgroups. 

## DISCUSSION

The current study comprehensively highlights how acidic and alcoholic oral environments adversely influence the physical and aesthetic characteristics of orthodontic thermoplastic materials. Yield strength, stiffness, and elastic modulus were all notably reduced following exposure, especially to carbonated and alcoholic beverages.

These findings corroborate those of Femina et al.,^13^ who demonstrated decreased tensile yield stress of thermoplastic aligners after immersion in carbonated solutions over a two-week period. Liu et al.^14^ similarly found that storage in artificial saliva and carbonated liquids decreased tensile strength and elongation, indicating molecular degradation due to acidic hydrolysis.

In the current analysis, EVA-based Bioplast showed significantly better mechanical performance than Erkoflex, corroborating the findings of Ryokawa et al.^15^, who reported that differences in EVA formulations yield variable mechanical resilience. The superior performance of CA Pro+ and Erkodur-Al in mechanical properties aligns with the findings by Agarwal et al.^16^, who emphasized that multilayered aligner materials demonstrate lower stress relaxation and superior mechanical stability over time. This is further supported by Jia et al.,^17^ who demonstrated that multi-layered aligners exhibit improved mechanical performance and force control, compared to single-layer designs. These material-specific advantages may also influence clinical outcomes, as reported by Kravitz et al.,^18^ who observed predictable tooth movement with Invisalign^®^, highlighting the importance of material properties in achieving effective orthodontic treatment.

Regarding stiffness, the present findings parallel the work of Hamid et al.^19^, in which PET-G-based sheets, like Duran and Erkodur, had higher rigidity, due to polymer chain configuration. The better force dissipation properties of Bioplast, despite also being EVA-based, suggest material formulation plays a crucial role, as confirmed by Coto et al.^20^, who attributed such properties to manufacturing-controlled viscoelastic behavior.

For elastic modulus, the present results affirm the conclusions of Nakornnoi et al.^21^ and Al Noor et al.^22^, who noted that thicker materials withstand higher elastic deformation before yielding, which has practical relevance when selecting materials for occlusal splints that bear functional loads.

Colour stability was compromised most in samples exposed to brandy. As ΔE values reflect the magnitude of colour change, they are inversely related to colour stability; thus, greater ΔE values indicate reduced stability. This is consistent with Pérez et al.^23^ and Taraboanta et al.^24^, who documented that alcohol-aged in oak barrels carries tannins and phenolics that penetrate thermoplastic surfaces and cause irreversible chromatic changes. Likewise, Aldweesh et al.^25^ emphasized that long-term exposure to acidic and pigmented solutions exacerbates discoloration in a time-dependent, material-specific manner.

Olteanu et al.^26^ observed greater staining in multilayered materials like CA Pro+ in acidic beverages, particularly cola and red wine, corroborating our observation of CA Pro+’s discoloration in carbonated drinks. Raffta Sánchez et al.^27^ further reinforced that low-pH beverages not only impair mechanical integrity, but also accelerate optical degradation, diminishing the clinical appeal and transparency of appliances. These findings are also consistent with Kobkiatkawin et al.^28^, who demonstrated that exposure to coffee, tea, and cola leads to clinically perceptible color changes in clear aligners, highlighting the role of beverage composition in staining susceptibility.

Although statistically significant differences in ΔE values were observed among groups, most values in the present study were below 1.0, which is considered imperceptible to the human eye. In color science literature, ΔE thresholds of 2.7-3.3 are generally accepted as the minimum for clinical perceptibility.^29^ The relatively short immersion protocol adopted in this study (10 minutes/day for 14 days) may have limited the extent of discoloration. Prolonged or continuous immersion could potentially yield higher ΔE values and more clinically evident color changes. Nevertheless, the statistically significant differences found within this limited exposure model emphasize that different thermoplastic materials exhibit varying susceptibility to staining.

The reduction in yield strength and elastic modulus observed in the beverage-exposed groups indicates possible degradation of thermoplastic material integrity. Similarly, noticeable color changes suggest surface alterations that could influence patient-perceived aesthetics over time.

These findings carry important clinical implications. The reduction in mechanical properties, such as yield strength and stiffness, may compromise the force levels delivered by thermoplastic orthodontic appliances, potentially affecting the predictability and efficiency of tooth movement. Changes in color stability can also impact patient satisfaction, particularly for adults seeking aesthetic, transparent aligners or retainers. Therefore, clinicians should consider material-specific responses to commonly consumed beverages and advise patients to minimize the exposure of thermoplastic appliances to acidic or alcoholic drinks, to maintain their mechanical integrity and aesthetic quality throughout treatment.

It should be noted that, *in vivo*, thermoplastic materials are continuously exposed to saliva, temperature fluctuations, mechanical stresses, and dietary acids throughout the day. The 10-minute daily immersion used in this study likely underestimates the cumulative effects of such exposure. Future investigations employing cyclic or prolonged immersion protocols could more closely approximate clinical conditions and provide a better understanding of long-term material degradation.

## LIMITATIONS

This *in-vitro* study has some inherent limitations. It does not fully replicate the oral environment, which includes saliva flow, temperature changes, enzymatic activity, mechanical forces, and plaque; therefore, *in-vivo* validation is required. 

The 10-minute daily immersion over 14 days simulates occasional beverage exposure, but likely underestimates cumulative changes during continuous long-term wear.

Only one carbonated drink (Thums Up) and one alcoholic beverage (brandy) were tested. Thums Up was chosen as the most widely consumed cola in India and can reasonably represent cola-type drinks in the region, while brandy was selected due to its frequent local consumption and relevance to regional drinking habits. However, different beverages may affect thermoplastic materials differently. Future studies should include a wider range of drinks and longer or cyclic exposure protocols to better approximate clinical conditions.

## CONCLUSION

The mechanical properties and colour stability of thermoplastic retainers, aligners, and occlusal splints are significantly affected by exposure to acidic and alcoholic beverages, with carbonated and alcoholic drinks causing the greatest reductions in yield strength, stiffness, and elastic modulus, while saliva preserved mechanical integrity. Among splints, Bioplast demonstrated superior mechanical performance, and aligners showed minimal color change. Retainers exhibited noticeable decreases in mechanical strength and slight discoloration following such exposure. Clinically, limiting contact of these appliances with acidic and alcoholic beverages helps maintain their durability, aesthetics, and overall effectiveness, supporting optimal orthodontic treatment outcomes.

## Data Availability

All data generated or analyzed during this study are included in this published article.
